# Fundamental population–productivity relationships can be modified through density-dependent feedbacks of life-history evolution

**DOI:** 10.1111/eva.12217

**Published:** 2014-10-08

**Authors:** Anna Kuparinen, Nils Christian Stenseth, Jeffrey A Hutchings

**Affiliations:** 1 Department of Environmental Sciences, University of HelsinkiHelsinki, Finland; 2 Centre For Ecological and Evolutionary Synthesis, Department of Biosciences, University of OsloOslo, Norway; 3 Department of Biology, Dalhousie UniversityHalifax, NS, Canada

**Keywords:** Atlantic cod, fisheries-induced evolution, overfishing, per capita population growth rate, recovery, recruitment

## Abstract

The evolution of life histories over contemporary time scales will almost certainly affect population demography. One important pathway for such eco-evolutionary interactions is the density-dependent regulation of population dynamics. Here, we investigate how fisheries-induced evolution (FIE) might alter density-dependent population–productivity relationships. To this end, we simulate the eco-evolutionary dynamics of an Atlantic cod (*Gadus morhua*) population under fishing, followed by a period of recovery in the absence of fishing. FIE is associated with increases in juvenile production, the ratio of juveniles to mature population biomass, and the ratio of the mature population biomass relative to the total population biomass. In contrast, net reproductive rate (*R*_*0*_) and per capita population growth rate (*r*) decline concomitantly with evolution. Our findings suggest that FIE can substantially modify the fundamental population–productivity relationships that underlie density-dependent population regulation and that form the primary population-dynamical basis for fisheries stock-assessment projections. From a conservation and fisheries-rebuilding perspective, we find that FIE reduces *R*_0_ and *r*, the two fundamental correlates of population recovery ability and inversely extinction probability.

## Introduction

Life histories can change over contemporary time scales owing to plastic and evolutionary responses to alterations in interspecific interactions (e.g., Reznick et al. 1997), environmental shifts (Merilä and Hendry 2014), as well as human-induced disturbances and anthropogenic selection generated by factors such as harvesting (Hendry et al. 2008; Darimont et al. 2009). Changes in key fitness-related life-history traits such as age and size at maturity, growth rate and adult body size will inevitably feedback to population dynamics, as they will affect rates of natural mortality and reproduction (Hutchings 2005, Saccheri and Hanski 2006; Kinnison and Hairston 2007). For example, in Soay sheep (*Ovis aries*), it has been shown that a substantial proportion of variation in population growth can be attributed to fluctuations in life-history traits (Coulson et al. 2006; Pelletier et al. 2007). The interplay between life-history evolution and population dynamics, which can be manifest by so-called eco-evolutionary dynamics, forms a fundamental link between evolutionary biology and population ecology (Kinnison and Hairston 2007; Palkovacs et al. 2012).

Density dependence is one of the key mechanisms responsible for regulating population dynamics. Population rebuilding potential, for example, is typically strongly subject to density-dependent rates of juvenile and biomass production: strongly negative density dependence in juvenile production will ensure that a sparse population recovers rapidly, whereas positive density dependence at a low abundance (i.e. an Allee effect; Stephens et al. 1999) can slow down or even impede recovery (Courchamp et al. 1999; De Roos and Persson 2002). From the perspectives of persistence and recovery of declined populations, density dependence offers a pathway through which life-history change can effectively modify population dynamics (Kinnison and Hairston 2007; Hendry et al. 2011). Drastic declines in population density can also give rise to life-history evolution, as suggested by the theory of evolutionary rescue: in populations that have fallen below a critical level of abundance, rapid adaptation of life histories can be a vital mechanism rescuing the population from extinction (Bell 2013; Ferriere and Legendre 2013; Carlson et al. 2014).

Trends in life histories towards earlier maturation at smaller body sizes have been recently documented in numerous commercially exploited fish populations (Hutchings and Baum 2005; Sharpe and Hendry 2009). In some cases, the magnitude of change appears to be positively correlated with the intensity of fishing, such that life-history modification has been hypothesized to reflect evolutionary responses to intensive, size-selective fishing (Devine et al. 2012; Audzijonyte et al. 2013). Namely, high adult mortality, often coupled with selective removal of large individuals, can lead to evolution towards earlier maturation at a smaller body size (e.g. Hutchings 2005, Law 2000; Heino and Godø 2002). While such fisheries-induced evolution (FIE) is not considered uncommon (Jørgensen et al. 2007; Hutchings and Fraser 2008; Heino et al. 2013), its impacts on population resilience and recovery ability have remained largely unexplored and, to some extent, controversial.

By definition, adaptation to the prevailing environment is reflected by an increase in average individual fitness relative to the fitness of other possible phenotypes (Roff 2002). In the context of FIE, adaptation might be reflected by life-history changes that optimize fitness in the presence of fishing. A recent meta-analysis by Neubauer et al. (2013) detected that fish populations exposed to moderate levels of overfishing for a comparatively long period of time had increased recovery ability when compared to populations subjected to overfishing for shorter periods of time. This was suggested to reflect evolutionary adaptation to fishing and its feedbacks on population productivity. In contrast, simulation studies on the recovery of Atlantic cod (*Gadus morhua*) suggest that FIE might reduce productivity as measured through recruit-per-spawner ratio (number of juveniles surviving to an age at which they first become vulnerable to fishing, divided by the spawning stock biomass; Enberg et al. 2009), but not demonstrably affect per capita population growth rates (Kuparinen and Hutchings 2012). The topic was also recently touched upon by Heino et al. (2013) who argued that FIE might at least initially increase per capita population growth rates.

In a fisheries context, density-dependent population productivity is commonly described as a function of biomass of mature individuals, the so-called spawning stock biomass (SSB; e.g. Myers 2001). Inspired by the contrasting findings and arguments surrounding the impacts of FIE on population growth and recovery ability, we investigate how relationships between SSB and alternative metrics of population productivity might be altered by FIE. To this end, we utilize an individual-based mechanistic simulation model parameterized for the northern Newfoundland stock of Atlantic cod (Kuparinen and Hutchings 2012) and simulate eco-evolutionary population dynamics over a period of fishing followed by a period of recovery. During the period of recovery, we compare (i) recruit production, (ii) recruit-per-spawner ratio, (iii) net reproductive rate (*R*_*0*_), and (iv) per capita population growth rate (*r*) as functions of SSB in the presence/absence of FIE.

## Methods

### Simulation design

Preadapted cod populations were first simulated for 100 years in an equilibrium state to ensure ecological and evolutionary stability, after which they were subjected to an instantaneous rate of fishing mortality, F, of 0.2 for another 100 years. Fishing selectivity depended on body size and followed a logistic selectivity curve e^−12.5 + 0.25 × length^/(1 + e^−12.5 + 0.25 × length^) appropriate for bottom trawling in Newfoundland waters (Myers and Hoenig 1997; see Supporting Information). The period of fishing was followed by a 100-year period of recovery in the absence of fishing. In our simulations, recruitment was quantified through the production of juveniles that survive up to age 3 (years) at which age they recruited to fisheries (Hutchings 2005). At each time step, we recorded several population metrics as well as life-history traits of individuals. Our particular emphasis was on SSB, total population biomass, recruitment and, for each cohort, *R*_0_ and *r*; the latter parameter was estimated through *r* = log(*R*_0_)/*T*, where *T* is the generation time approximated by the average age of the spawning population during the lifetime of the cohort. Simulations were repeated with model versions that either incorporated or excluded life-history evolution during and after fishing (20 replicated runs for each scenario).

### Model and its parameterization

Although the simulation model has been described extensively elsewhere (Kuparinen and Hutchings 2012; Kuparinen et al. 2012), below we provide a summary of the model features and its parameterization. The model describes individual life histories through Von Bertalanffy (VB) growth trajectories *L*(t) = *L*_*∞*_−(*L*_*∞*_−*L*_*0*_)e^−*k*t^, where t is the age of a fish, *L*(*t*) is the length of a fish at age *t*, *L*_*∞*_ is the asymptotic body length, *L*_0_ is the average length at *t* = 0 and *k* is the growth parameter that describes the rate at which an individual reaches its *L*_*∞*_ (von Bertalanffy 1938). Parameters *k* and *L*_*∞*_ as well as *L*_*∞*_ and the length at maturity are known to be strongly correlated with one another (Charnov 1993), such that they jointly produce a ‘life-history type’. We modelled the inheritance of life-history types although additive effects of 10 independent loci, two alleles at each (coded with 0 and 1). In the evolving model version, alleles were passed from parents to juveniles following normal Mendelian inheritance. In the nonevolutionary simulations, alleles for each juvenile were drawn from a fixed pool that was recorded during the 30 years prior to the beginning of fishing (and, thus, reflected genetic diversity at equilibrium conditions; see results section below).

In both evolving and nonevolving simulations, VB parameters for each juvenile were derived based on the sum of the allele values, which ranged between 0 and 20. The allele sum was linearly translated to the value of *L*_*∞*_ at a range from 30 to 130 cm. A random number drawn from normal distribution with mean 0 and standard deviation (SD) 3.5 was added to the allele sum value to yield realistic heritabilities for the realized life histories (∼0.2–0.3, Mousseau and Roff 1987). Robustness of the results to the chosen value of this parameter was explored by repeating the simulation design with SD values of 4.5 and 2.5, which yielded very low and high heritabilities, respectively (see Supporting Information). The value of k was then predicted based on the value of *L*_*∞*_. To parameterize the relationship between *L*_*∞*_ and k, we utilized empirically estimated cod growth trajectories measured from otoliths collected from a land-locked cod population on Baffin Island, northern Canada (Hardie and Hutchings 2011). The key advantage of using data from this population is that it is unexploited and thereby reflects natural phenotypic diversity of life histories in a cod population in the absence of human-induced selection. In terms of VB parameters and length at maturity, the population is also very similar to marine cod populations at northern latitudes (e.g. Northeast Arctic cod, northern cod; Kuparinen and Hutchings 2012). VB growth curves were first fitted to each empirically measured length-at-age trajectory through nonlinear regression, and the relationship between the estimated *L*_∞_ and log-transformed k parameters was then estimated based on linear regression, such that the estimated model was log(*k*) = −0.609−0.013 × *L*_∞_ (with residual standard error of 0.305). The length–weight relationship (weight = 3.52 × 10^−6^ × length^3.19^) was also estimated from the same empirical data (Kuparinen et al. 2014).

At each time step, the processes of natural mortality, growth, maturation and reproduction were simulated on an individual basis. Baseline (instantaneous) natural mortality was assumed to be 0.12 in addition to which a survival cost of reproduction of 0.10 was added for mature individuals. These parameters were derived by calibrating the simulation model to provide the closest match with the empirically estimated length-at-age trajectories (Kuparinen et al. 2014). Density dependence of growth was implemented such that within one time step (year), an individual progressed along its VB trajectory according to a time increment of Δt = e^15−17.6 × c^ (1+e^15−17.6 × *c*^)^−1^, where *c* is the ratio of population biomass to carrying capacity (K). While the exact mathematical formulation is somewhat arbitrary, the overall ability of the model to predict the empirically observed cod life histories under equilibrium conditions was ensured by comparing model outputs with the empirical observations (as we did with the mortality parameters). Individuals whose body length exceeded 66% of *L*_*∞*_ were considered mature (Jensen 1997). All mature individuals reproduced at each time step, and each mature female was randomly assigned to a mature male. Recruit production depended on female body size and SSB. We utilized an estimate of egg production (eggs = (0.48 × ((female weight + 0.37)/1.45) + 0.12)×10^6^) measured for northern cod in the early 1960s (May 1967, Hutchings 2005), when the population abundance is considered to have been approximately 40% of its K and SSB was estimated to be about 1.4 million tonnes. Therefore, we scaled the egg production up or down depending on whether the SSB in the simulations was below or above 0.4 × K. The scaling was based on the relative increase/decrease in recruitment in the Beverton-Holt stock–recruitment relationship for northern cod (recruits = 1060*SSB/(1 + SSB/1900); Myers et al. 1995). The final egg production ranged between 174% (at *SSB*≈0) and 61% (at *SSB*≈K) of that given by the egg production equation above. Survival from birth until age 3 years was set to 1.13 × 10^−6^ (Hutchings 2005). For further details, see Kuparinen and Hutchings (2012) and Kuparinen et al. (2012).

## Results

During the 100-year period of fishing, population biomass declined to approximately 3.7% of K in both the evolutionary and nonevolutionary scenarios. After the end of fishing (year 200), biomass began to rebuild such that it reached an equilibrium within 40–50 years. At equilibrium, realized heritability of life-history type ranged between 0.26 and 0.29. In the evolutionary simulations, fishing generated life-history evolution towards lower *L*_*∞*_ and, thus, younger age and smaller size at maturity. By the end of the fishing period, the average *L*_*∞*_ had declined from 80.6 to 64.7 cm, the average age at maturity from 7.3 to 4.6 years, and the average length at maturity from 53.4 to 42.0 cm. In the nonevolutionary simulations, age at maturity decreased during the fishing period (from 7.3 to 5.4 years) because of density-dependent feedbacks, but it returned to its prefishing level concomitantly with the rebuilding of stock biomass to equilibrium levels. Reversal of evolutionary change was very slow, such that during the first 100 years of recovery, *L*_*∞*_ increased only by 2.7 cm, age at maturity by 0.5 years and length at maturity by 3.6 cm. In the following section, we focus on the rebuilding period that followed the end of fishing and compare how the productivity of populations that had experienced FIE differed from those of populations that had not evolved during fishing.

**Figure 1:**
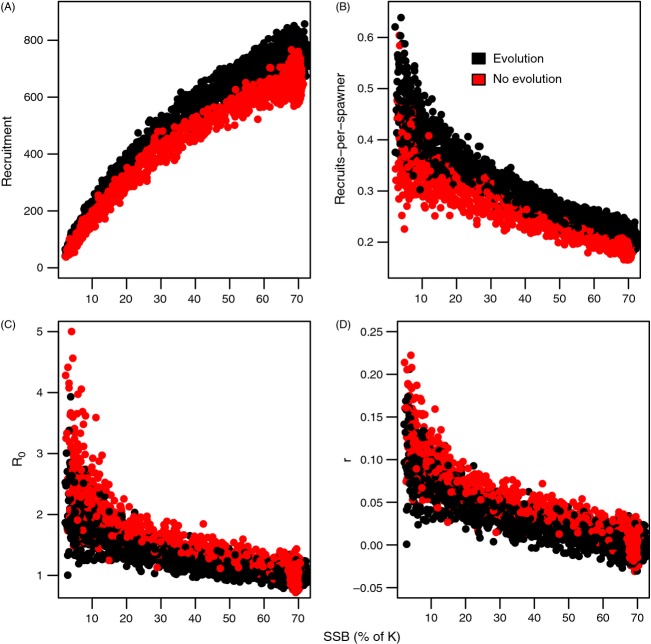
Productivity of a population as a function of spawning stock biomass (SSB) described through (A) total number of recruits produced, (B) recruit-per-spawner ratio (recruits/SSB), (C) net reproductive rate (*R*_0_) and (D) per capita population growth rate (*r*) per unit of time. SSB is expressed as the proportion of population carrying capacity (K). Evolutionary and nonevolutionary scenarios are indicated with colours. For panels c and d, SSB in the year a cohort is born is plotted against the *R*_0_ and *r* values that were calculated over the lifetime of the given cohort. Values are drawn from 20 replicated simulations for both evolving and nonevolving scenarios.

At a given SSB level, both the total number of recruits and the recruit-per-spawner ratio were higher in the evolved populations when compared to the nonevolved populations (Fig. 1A, B). In contrast, a reverse shift could be seen in the net reproductive rate (*R*_*0*_) and per capita population growth rate (*r*). Plotted against the SSB at the year the cohort was born, *R*_0_ and *r* were higher in the nonevolved populations when compared to the evolved populations (Fig. 1C, D). The differences in recruit-per-spawner ratio, *R*_0_ and *r* were most pronounced at very low SSB levels (Fig. 1). The patterns shown in Fig. 1 were the same when total biomass was used as a proxy of population's reproductive capacity instead of SSB, and this was plotted on *x*-axis.

**Figure 2:**
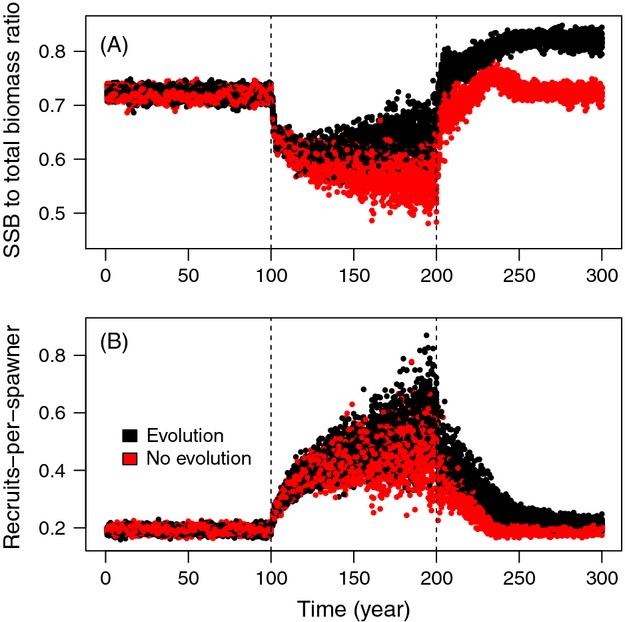
Temporal development of (A) the proportion of spawning stock biomass (SSB) of the total population biomass and (B) the recruits-per-spawner ratio across the simulation period (300 years). The beginning (year 100) and the end (year 200) of fishing are indicated with dashed vertical lines. Evolutionary and nonevolutionary scenarios are indicated with colours. Values are drawn from 20 replicated simulations for both evolving and nonevolving scenarios.

Differences in recruit production between evolved and nonevolved populations could be explained through differences in the overall population biomass at a given SSB level: the ratio of SSB to total population biomass started to increase along with fisheries-induced evolution and the ratio remained higher after the end of fishing (Fig. 2A) because of differences in life histories. In practice, this means that at a given level of SSB, an evolved population was sparser (i.e. existed at a lower density) than a nonevolved population. Therefore, individual growth was faster in an evolved population (less density-dependent regulation on growth), leading to higher juvenile production at a given level of SSB (Fig. 2B).

The results were highly robust to the heritability of life-history types. Despite considerable differences in the magnitude of heritability between the alternative parameterizations, evolutionary responses to the fishing period were very similar and, thus, the patterns seen in the stock–productivity relationships were analogous to those described above (see Supporting Information).

## Discussion

Exploration of eco-evolutionary dynamics has proven to be useful to identify and understand ecological feedbacks of evolutionary processes and to project the role of evolution in the applied contexts of conservation and harvesting (Pelletier et al. 2007; Coulson et al. 2010; Schoener 2011; Palkovacs et al. 2012). The present study shows that contemporary evolution in fish life histories can alter fundamental population–productivity relationships and, therefore, constitutes an underlying mechanism of new ‘productivity regimes’. Such overall changes in productivity have been documented in numerous commercially exploited fish stocks (e.g. Vert-pre et al. 2013). Additionally, we found that SSB may not be as good a proxy of population abundance as traditionally considered in the fisheries context (Hutchings et al. 2010). FIE increases the ratio of SSB to total biomass – a finding also supported by earlier eco-evolutionary analyses (e.g. Enberg et al. 2010). Consequently, after FIE has occurred, the total biomass is lower than what had been anticipated based on an observed/estimated level of SSB. Such overestimation of population size can give rise to overly optimistic prospects of population recovery and lead to overfishing.

Maximum per capita population growth rate (*r*_max_) is considered a universal correlate of recovery ability and, inversely, risk of extinction (Dulvy et al. 2004; Mace et al. 2008). In the context of fisheries research, population growth ability is typically approximated by recruit-per-spawner ratio, but the present study suggests that this can be severely misleading. The impacts of FIE on recruit-per-spawner and on *r* were found to be opposite. Both recruit production and recruit-per-spawner ratios increased in the presence of fisheries-induced evolution (FIE) (Fig. 1A, B), whereas net reproductive rate and per capita population growth rate decreased (Fig. 1C, D). One mechanism underlying these shifts is that FIE increases the proportion of mature biomass in the population (Fig. 2), such that at a given mature biomass level (level of SSB), an evolved population is sparser, allowing individuals to grow faster than they would at the same SSB in a nonevolved population because of the latter's greater overall density. In terms of the annual juvenile production, even though FIE resulted in smaller mature fish, such that their individual reproductive output was lower (result not shown), this was compensated for by their larger number. However, lifetime reproductive output nonetheless declined, as earlier maturation led to higher mortality owing to the survival costs of reproduction (Bell 1980, Hutchings 2005, Kuparinen et al. 2014). Our findings therefore suggest that demographic costs associated with fisheries-induced selection can readily outweigh potential fitness gains involved with the adaptation of life histories, such that FIE might not be a sufficient mechanism to rescue an exploited population from extinction or to aid its rebuilding.

As with any modelling study, the results presented here are subject to numerous assumptions. As our modelling approach has been previously published and discussed elsewhere (Kuparinen and Hutchings 2012; Kuparinen et al. 2014), here, we focus on the assumptions and model features relevant for interpreting the findings of the present study. In this respect, the ways in which population abundance is assumed to affect demographic processes are of fundamental importance to understanding how, at a given level of mature biomass, evolution changes population dynamics. In our modelling approach, population density affected individual growth and recruitment. However, as traditionally assumed in fisheries contexts, recruitment was assumed to be only affected by mature biomass and, therefore, at a given SSB level, the density effect was the same for both evolved and nonevolved populations. In contrast, each individual's progress along its growth trajectory depended on the ratio of population biomass (both mature and immature) to carrying capacity, such that in the evolved populations, the progress along the growth curve was faster at a given SSB level. In reality, total biomass to carrying capacity ratio could also affect other processes such as natural mortality or juvenile production and, therefore, our results can be viewed to be conservative. Size of the mother was only assumed to affect the number of eggs produced but not their quality. In the evolved populations, reproducing fish were smaller and, consequently, our estimate of the upward shift in the stock-recruitment and recruit-per-spawner relationships might be overly optimistic. On the other hand, if larger fish do markedly better due to increased juvenile survival and decreased mortality, then the downward shifts seen in *R*_0_ and *r* due to FIE should be larger than those suggested by our results. Such differences might also explain why Enberg et al. (2010) did not find FIE to affect the SSB–recruitment relationship. They did not account for survival costs of reproduction but assumed natural mortality to decline as a function of body size, such that life-history composition of the spawning stock and its reproductive output might have been very different.

One interesting feature of our results is that the discrepancy in *R*_0_ and *r* between the evolutionary and nonevolutionary scenarios is greatest at low abundances, that is at low SSB levels. While the correlations between SSB and *R*_0_ or *r* still remain negative, the slopes clearly become smaller in the evolved populations; this indicates a weakening of the compensatory dynamics at a low abundance. Potentially, FIE could act as one underlying component that, together with other factors and processes taking place at low abundances, can bring about a demographic Allee effect (see Stephens et al. 1999; De Roos and Persson 2002). Many overfished stocks that potentially have also experienced FIE have shown unexpectedly little evidence of recovery despite reductions in fishing pressure (Hutchings and Reynolds 2004, Neubauer et al. 2013), suggesting that some unaccounted factors can limit population growth at low abundances. For example, in Atlantic cod demographic Allee effects have been documented in several stocks (Keith and Hutchings 2012). From the perspective of eco-evolutionary dynamics, it becomes an interesting question to identify conditions under which changes in fish life-history traits could bring about a demographic Allee effect and, thereby, substantially increase the risk of extinction and limit population recovery ability (see De Roos and Persson 2002).

The present study illustrates the ability of contemporary life-history evolution to modify the basic ecological dynamics of a population and affect its renewal ability and reproductive capacity. As shown here in the fisheries context, accounting for evolutionary changes in ecological properties and processes is vital to reliably predict population development in the future, assess the conservation status of the population and establish limits for sustainable harvesting (e.g. Kinnison and Hairston 2007). Moreover, eco-evolutionary changes can also underlie demographic Allee effects (De Roos and Persson 2002) and account for productivity regime shifts (e.g. Pelletier et al. 2007) that, in the fisheries context, have been traditionally assumed to be caused by the environment (Vert-pre et al. 2013). These examples suggest that future research is needed to investigate to what extent ecological properties of harvested fish populations could indeed be explained or modified through their evolutionary history (see Neubauer et al. 2013). To this end, eco-evolutionary simulations provide a powerful framework to explore the interplay of ecological and evolutionary processes in both fundamental and applied contexts (e.g., Laugen et al. 2014).

## Supporting Information

**Figure S1.** Relative selectivity of fishing as a function of fish body length.

**Data S1.** Sensitivity to the magnitude of heritability.
