# Supplementary figures and images for: Fundamental population–productivity relationships can be modified through density-dependent feedbacks of life-history evolution
Source: Evol Appl. 2014 Oct 8;7(10):1218–25. doi: 10.1111/eva.12217 (PMC4275093; doi:10.1111/eva.12217)

**Fig. 1** Relative selectivity of fishing as a function of fish body length.


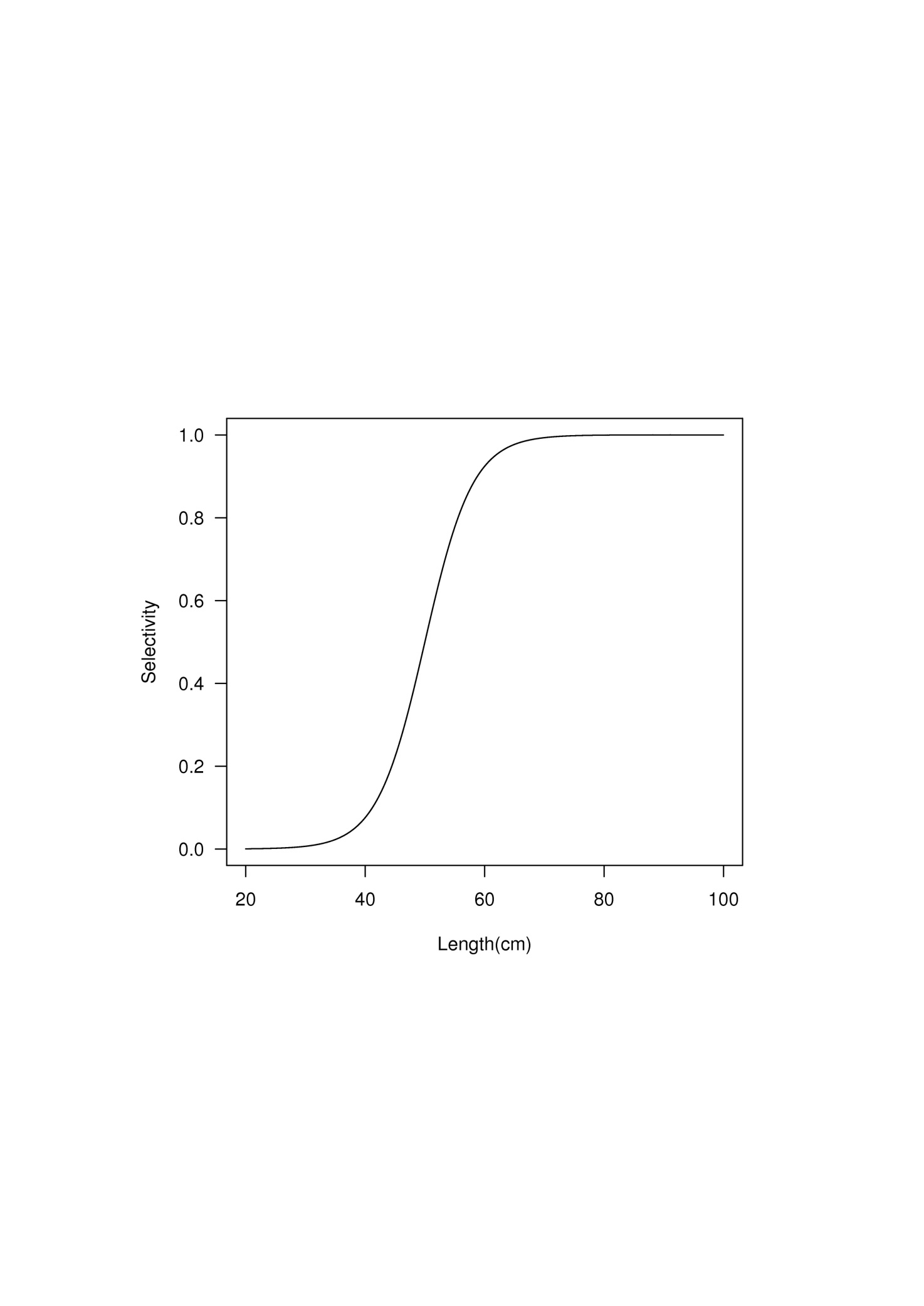

Supplement: Supplementary file 1 — Figure S1. Relative selectivity of fishing as a function of fish body length. [file eva0007-1218-sd1.docx]
