# Supplementary material for: Fundamental population–productivity relationships can be modified through density-dependent feedbacks of life-history evolution
Source: Evol Appl. 2014 Oct 8;7(10):1218–25. doi: 10.1111/eva.12217 (PMC4275093; doi:10.1111/eva.12217)
Supplement: Supplementary file 2 — Data S1. Sensitivity to the magnitude of heritability. [file eva0007-1218-sd2.docx]

**Sensitivity to the magnitude of heritability**

To explore the robustness of the results to the chosen amount of phenotypic variation about the genetic trait value, we repeated the simulation design with low and high heritability scenarios. To produce low or high heritabilities for the life-history types, the standard deviation of the normal distribution from which random noise on the top of allele sum was generated was set to 4.5 or 2.5, respectively. The realized heritabilities for the low scenario ranged between 0.18-0.20 whereas for the high scenarios the range was 0.41-0.44. Despite the large difference in the magnitude of the heritability, after 100 years of fishing the evolutionary responses to fishing were very similar, such that L_∞_ declined from the initial 80 cm to about 66 cm in the low heritability scenarios and to 64 cm in high heritability scenarios. Consequently, patterns in the stock-productivity relationships were very similar (Fig 1, Fig 2) and provided a close match with those derived under realistic heritabilities.

**
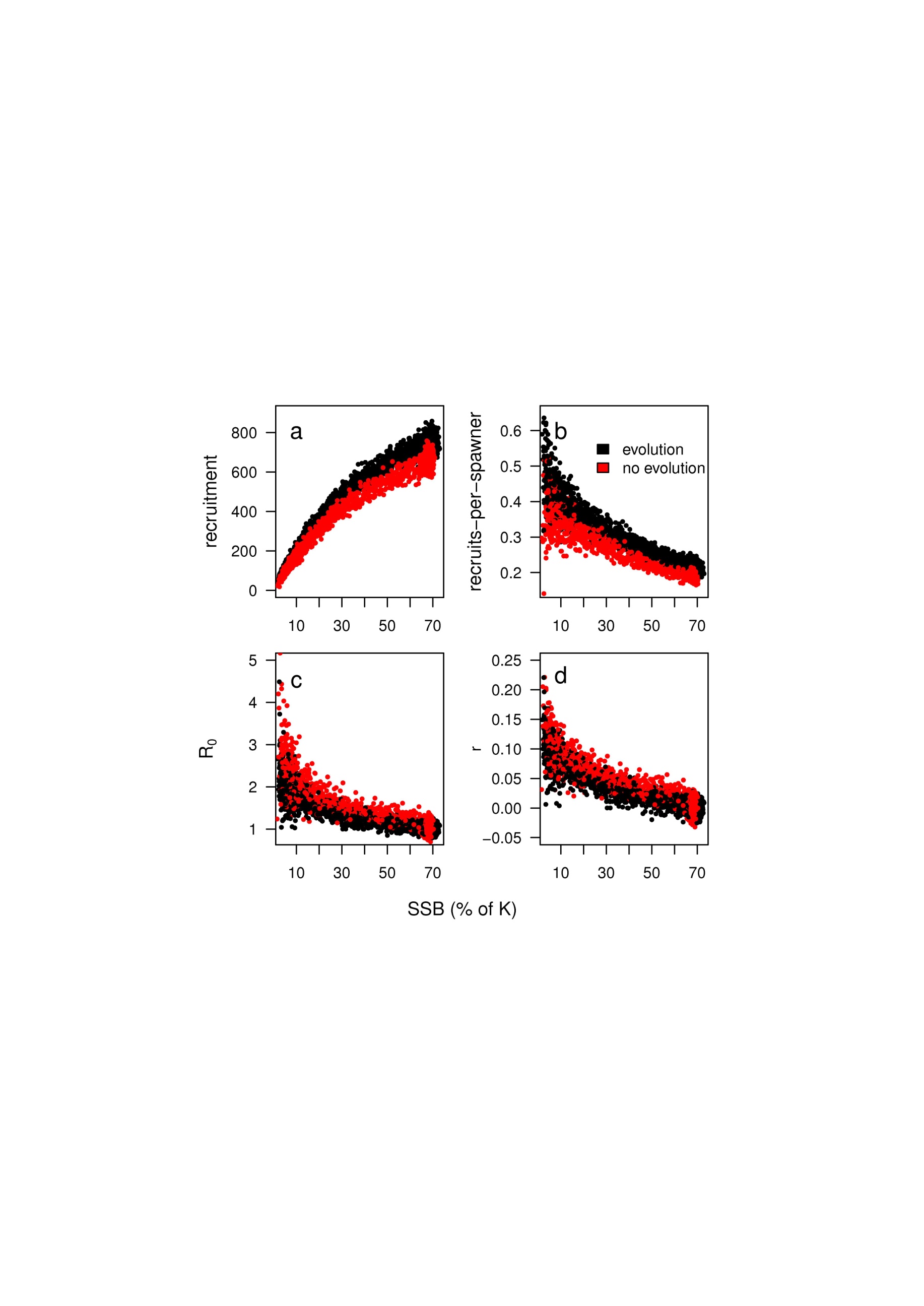
Fig. 1** Simulations in the presence of low heritability for life-history types: Productivity of a population as a function of spawning stock biomass (SSB) described through a) total number of recruits produced, b) recruit-per-spawner ratio (recruits/SSB), c) net reproductive rate (*R_0_*), and d) per capita population growth rate (*r*) per unit of time. SSB is expressed as the proportion of population carrying capacity (K). Evolutionary and non-evolutionary scenarios are indicated with colours. For panels c and d, SSB in the year a cohort is born is plotted against the *R_0_* and *r* values that were calculated over the lifetime of the given cohort. Values are drawn from 20 replicated simulations for both evolving and non-evolving scenarios.

**
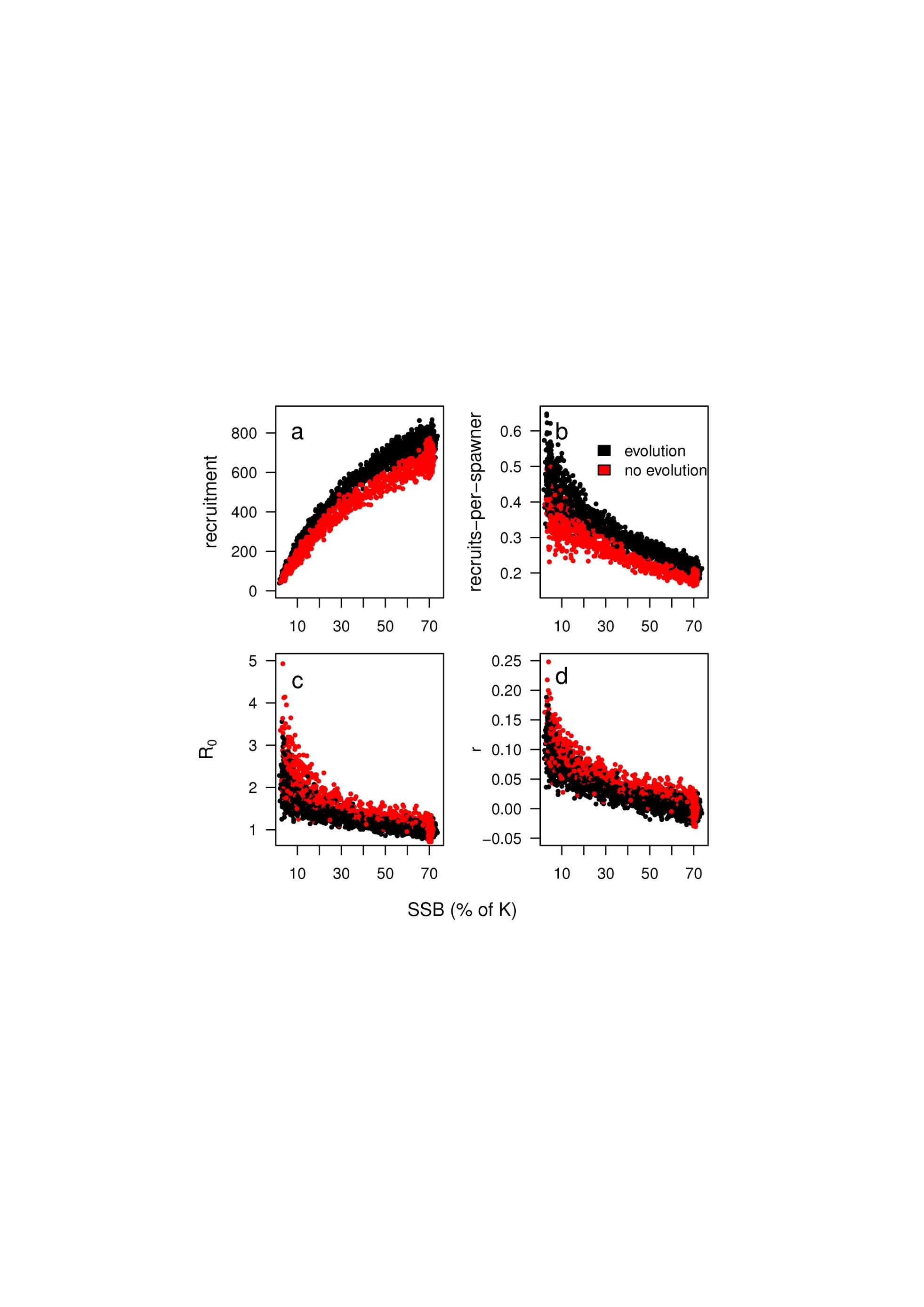
Fig. 2** Simulations in the presence of high heritability for life-history types: see legend of Fig. 2 for panel and colour explanations.
